# Exploring factors that influence the behavioural intention of medical students to use 3D gastroscopic model to learn how to operate gastroscope using UTAUT Model

**DOI:** 10.1186/s12909-023-04532-6

**Published:** 2023-08-07

**Authors:** Shuting Wei, Pu Ge, Jinzi Zhang, Shuxian Xu, Yujia Wang, Qiyu Li, Bojunhao Feng, Wenli Yu, Baojun Suo, Yueyang Zhang, Mingxing Wang, Xinying Sun, Zhiqiang Song, Yibo Wu

**Affiliations:** 1 Translational Medicine Research Center, Medical Innovation Research Division, Fourth Medical Center of the Chinese PLA General Hospital, Beijing, China; 2 School of Traditional Chinese Medicine, Beijing University of Chinese Medicine, Beijing, China; 3 College of Humanities and Social Sciences, Harbin Medical University, Heilongjiang, China; 4 China Pharmaceutical University, Nanjing, China; 5 School of Humanities and health management, Jinzhou Medical University, Jinzhou, China; 6 School of Medicine, Macau University of Science and Technology, Macao, China; 7 School for Sports Humanities and Social Science, Jilin Sport University, Changchun, China; 8 Department of Gastroenterology, Peking University Third Hospital, Beijing, China; 9 Department of Epidemiology, State Key Laboratory of Cardiovascular Disease, Fuwai Hospital, National Center for Cardiovascular Diseases, Chinese Academy of Medical Sciences and Peking Union Medical College, Tsinghua University, Beijing, China; 10 The Third Clinical Medical College, Harbin Medical University, Heilongjiang, China; 11 School of Public Health, Peking University, Beijing, China

**Keywords:** Unified theory of Acceptance and Use of Technology, Structural equation modeling, Virtual reality, Gastroscopy, Behavioural intention

## Abstract

**Background:**

The application of virtual reality (VR) in gastroscopic operation teaching can be safe and effective, but the advantages can be realized only when students accept and use it. This study aims to identify the factors influencing Chinese clinical medical postgraduates on their intention to use the 3D gastroscopic model constructed based on VR technology using Unified Theory of Acceptance and Use of Technology (UTAUT) model. Students’ demographic factors are also taken into consideration.

**Methods:**

All methods were carried out in accordance with relevant guidelines. Data were collected from clinical medical postgraduates students in China using stratified sampling. A total of 292 questionnaires including valid responses were used in this study. Data were processed using Amos 24.0 and SPSS 26.0 software and the statistical analysis technique was based on structural equation modeling (SEM).

**Results:**

The results showed that different from the mediator of home location and year of clinical learning, mediator of gender, university kind and graduate degree did not affect the behavioral intention. In addition, performance expectancy, facilitating condition, and social influence directly and indirectly have effect on behavioral intention. Also, the significance between social influence and performance expectancy, social influence and effort expectancy were verified.

**Conclusions:**

This study manifested that the proposed framework based on the UTAUT had explanatory power to identify the factors influencing the students’ behavioral intention to use the 3D gastroscopic model constructed based on VR technology. Whereas, an important variable of effort expectancy in the frame of the SEM were not certified, thereby indicating that particular attention should be paid to this variable by universities and teachers before applying 3D gastroscopic model constructed based on VR technology in teaching. Added preparatory work is required such as explaining the basic knowledge of the operating steps of VR model and make students adequately understand its accessibility, which can probably improve the intentions of them to use it. The positive effects of social influence on performance expectancy and effort expectancy we proposed was also verified in this study, which provided a direction for future research.

## Introduction

Gastroscopy is an important diagnostic and therapeutic tool used to assess and treat gastrointestinal disorders [1, 2]. Gastroscopy uses a thin, flexible tube that is inserted into the stomach, allowing the endoscopist to look directly at the stomach lesions. It is the method of choice for examining gastric lesions and is one of the techniques that must be mastered by gastroenterologists [3].

However, gastroscopy is a complex procedure that requires a high level of medical skill of the clinician. The traditional mode of medical education of using gastroscopy requires trainees to participate in the diagnosis and treatment of patients under the guidance of experienced preceptors, in which process, they continue to accumulate and improve their knowledge reserve and operational skills. For preceptors, it is difficult to take into account the teaching of trainees while ensuring the smooth operation, which not only affects the quality of clinical teaching but also increases the risk for patients [4]. For trainees, it is difficult to master gastroscopy operation technology, which may not only blow their confidence, but also cause pain to patients [5], let alone the possible medical risks [6, 7].

The history of using simulation technology to train gastroscopy operation dates back to the 1860s, representing a transition from traditional “hand to hand” endoscopy training to simulator-assisted teaching [8]. Over time, the gastroscopy simulator has evolved to utilize virtual reality (VR) technology to enhance its effectiveness in endoscopic training [9].

Virtual reality (VR) is defined as the use of computer modeling and simulation to enable a person to interact with an artificial three-dimensional visual or other sensory environment [10]. The integration of VR technology with clinical practice has significantly benefited medical education and experimentation [10]. The application of VR in gastroscopic operation teaching has provided trainees with a highly realistic diagnosis and gastroscopic environment, in which trainees can safely and effectively conduct all-round training and improve their orientation cognition, hand-eye coordination and operation ability [11]. Additionally, virtual reality gastroscopy simulators offer several advantages such as reducing risks and potential medical disputes, offering high repeatability, increasing practice opportunities, saving training time and costs, establishing training records, and developing individualized training plans [12]. Furthermore, these simulators have objective assessment functions that enable the evaluation of operator proficiency [13]. For instance, Qianru Wang et al. explored the excellence of VR animation technology for surgery, research, training and education using a gastroscopy simulation system as an example [11]. Similarly, Heather Lesch, et al. found that VR simulators like Toolkit for illustration of Procedures in Surgery (TIPS) can enhance trainees’ confidence in reproducing the steps of the procedure and serve as a valuable learning strategy [14].

Currently, the application of VR gastroscopy simulator in clinical training has been well-established internationally. In foreign countries, endoscopy training has successfully transitioned from traditional “hands-on” teaching to VR simulator-assisted teaching. However, in China, VR simulator-assisted teaching for gastroscopy is still in its early stages [8]. With the introduction of a series of new technologies, equipment and software like VR is expected to bring new opportunities to clinical medical practice, the development of VR-based gastroscopy teaching simulation technology in China is seen as the future trend of development.

## Theoretical basis and research hypotheses

Prior to the development of the Unified Theory of Acceptance and Use of Technology (UTAUT), there were numerous theoretical models of individual acceptance of information technology, but many of these models lacked comprehensive explanatory power, making it challenging for researchers to select the most suitable model based on their specific requirements. Venkatesh et al. addressed this issue by integrating eight widely accepted models at the time: Theory of Reasoned Action (TRA), Technology Acceptance Model (TAM), Motivational Model (MM), Theory of Planned Behaviour (TPB), Integrated Theory of Planned Behaviour and Integrated Technology Acceptance Model (C-TAM), Model of PC Utilization (MPCU), Innovation Diffusion Theory (IDF), and Social Cognitive Theory (SCT). This integration resulted in the creation of the Unified Theory of Acceptance and Use of Technology (UTAUT) [15] .

The model consists of four independent variables: Performance Expectancy (PE), Effort Expectancy (EE), Social Influence (SI) and Facilitating Condition (FC); Additionally, the UTAUT model includes four moderating variables, which are gender, age, experience, and voluntary use [15]. Performance expectation refers to the level of expectation of the users of the new technology for the technology or system; effort expectation refers to the cost in terms of time, manpower and intelligence if the user of the new technology has to spend in order to become proficient in the technology; social influence refers to the level of the role of the surrounding environment including interpersonal relationships, social environment, etc. on the users of the new technology; facilitating conditions refers to the knowledge base the users have about the new technology and the level of support from the relevant organizations for users to use the technology [15] .

Researchers have tested the model through numerous empirical studies and found that UTAUT has higher explanatory power than previous models [16]. The UTAUT model has been applied in many studies on the willingness to use and behaviour of new technologies since it was proposed. For example, Cilliers L et al. used the UTAUT model to study students’ acceptance of mobile phone use to seek health information [17]; Dwivedi YK and Hoque R et al. used it to study m-health applications [18, 19]. Although UTAUT model has been used in the field of virtual reality and education [20–22], there are no relevant studies that have utilized the UTAUT model to explore the intention to use the 3D gastroscopic model constructed based on VR technology, which implies that there is a research gap in this field.

The UTAUT was developed by integrating four constructs (performance expectance, effort expectancy, social influence, and facilitating conditions) to play an important role as direct determinants of users’ behavioural intention and using behaviour [23] According to Venkatesh et al. [15], performance expectance, effort expectancy and social influence have a significant effect on users’ behavioural intention. Consistently, we put forward hypotheses H3-H5 in this research. In 2012, Venkatesh et al. [24] extended the UTAUT model by hypothesizing and verifying that facilitating conditions also have a significant effect on users’ behavioural intention, which is consequently the basis of the hypothesis H6 of our study. In addition, several studies have demonstrated the positive effects of performance expectance, effort expectancy, social influence, and facilitating conditions on behavioural intention in the UTAUT model [25, 26]. Chien-wen Shen et al. studied the behavioural intention to use virtual reality technology in learning based on the UTAUT model, and the results showed that all four main factors in the UTAUT model positively influenced the behavioural intention to use virtual reality in learning [25]. In exploring the factors influencing the implementation of online hospitals and the adoption of mHealth services, Wang et al. concluded that performance expectations, effort expectancy, and facilitating conditions had a positive impact on patients’ willingness to use online hospitals [26].

Social influence is defined as the degree to which an individual perceives that important others believe he or she should use the new system [15]. If students’ important others such as teachers or classmates recommend them to use the 3D gastroscopic model constructed based on VR technology, they will probably consider the reasons of their recommendations-what are the advantages of this technology. Either the technology is easy to use, or will help them to attain gains in study performance. Therefore, outside the standard and extended UTAUT model, we put social influence as an antecedent variable and assumed that it would have a significant effect on performance expectance and effort expectancy, which are H1 and H2. Paula Philippi et al. validated and adapted the UTAUT to digital health based on the UTAUT model, in this study the authors proposed and tested the hypothesis of a correlation between the main factors (performance expectance, effort expectancy, social influence, and facilitating conditions) [27]. In the study of exploring the factors that influence college students’ intention to consistently use online course platforms Mengting Chen et al. combined the original UTAUT model, added new factors to propose a framework model and hypothesized a positive relationship between social influence and performance expectations [28]. Given that few previous studies have explored and verified the relationship between social influence and performance expectancy or effort expectancy, the hypothesis proposed in this study is innovative. Meanwhile the verification of the hypotheses will provide new directions for future research and provide more reference value for the practical application of the 3D gastroscopic model constructed based on VR technology.

Venkatesh et al. [15] presented four manipulated variables (gender, age, experience, voluntariness of use) that moderating the relationship between four latent variables and behavioural intention. The demographic sociological characteristics in this study were gender, types of universities, grade, year of clinical learning and home location. In consideration of the fact that the traditional high simulation devices are very expensive and unable to be used widely in medical schools [29], namely many of the respondents in this survey didn’t have the experience of using VR technology to learn, the use behaviour and the effect of experience is not considered in this study. Thus the moderating variable “experience” as well as the latent variable “use behaviour” were excluded from the model. In addition, almost all users are not forced to use the system, so the moderating variable “voluntariness” is excluded from the model. As for manipulated variable “age”, considering that most of the students in the study are in the same range of age and do not have very age difference, another demographic characteristic “year of clinical learning” was chosen as the moderating variable. The replacement of manipulated variables is another important innovation point of us. For a certain research group, the manipulated variables of the model may not be fully applicable, and the appropriate replacement according to the actual situation can increase the degree of adaptation of the model, which provides reference value for future research.

According to above mentioned, we hypothesized as follows:

H1: Social Influence positively affects Performance Expectancy .

H2: Social Influence positively affects Effort Expectancy .

H3: Performance Expectancy positively affects Behavioural Intention.

H4: Social Influence positively affects Behavioural Intention .

H5: Effort Expectancy positively affects Behavioural Intention .

H6: Facilitating Condition positively affects Behavioural Intention .

**Fig. 1:**
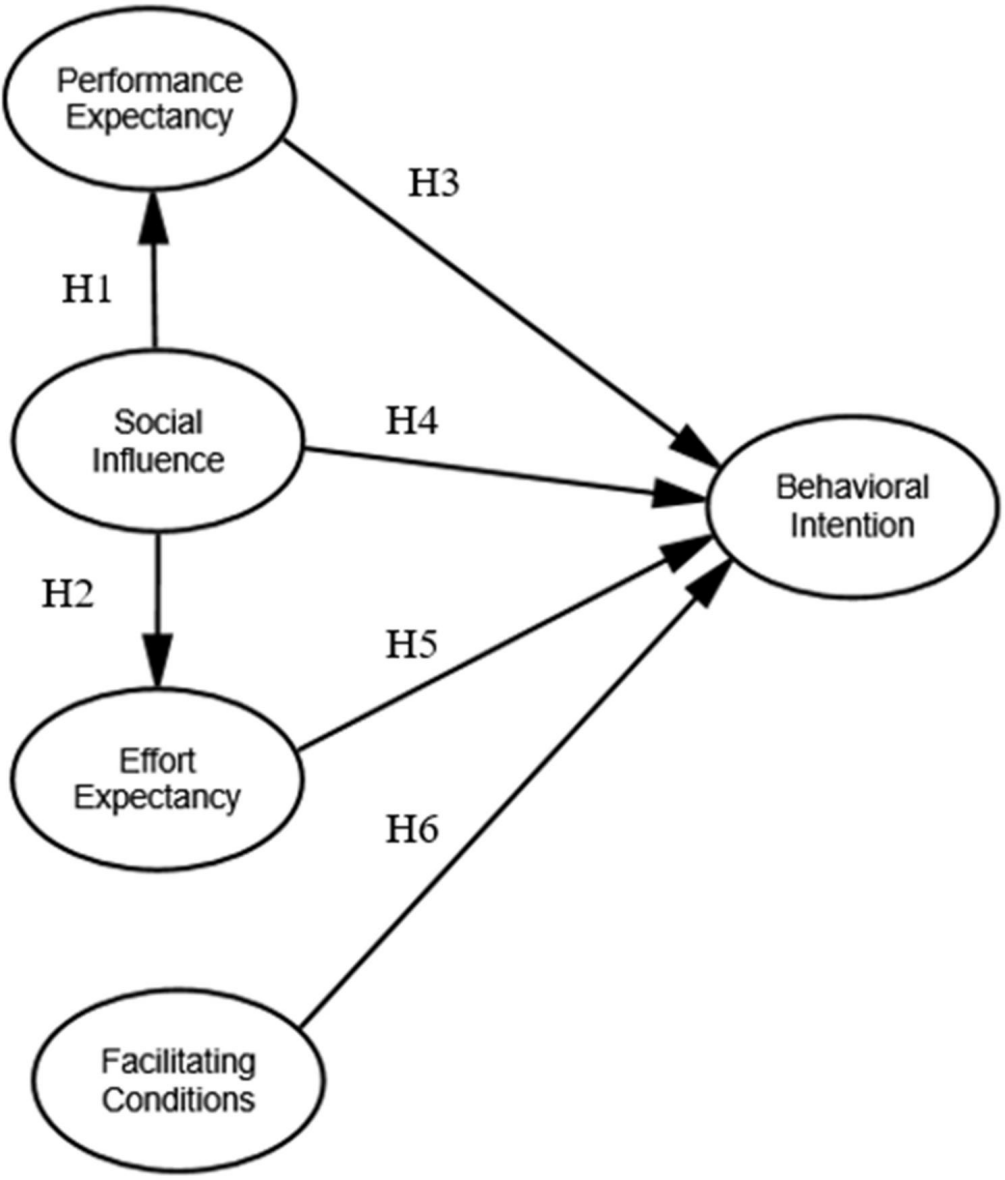
Research framework

Six hypotheses were shown in Fig. 1.

In this study, we will explore the four influencing factors of Chinese clinical medical postgraduates on their intention to use 3D gastroscopic model to learn to operate gastroscope based on UTAUT. Additionally, we will try to verify two hypotheses of the significant positive influence of social influence on performance expectancy and effort expectancy. Besides, we will analyze the influence of students’ demographic factors on the constructs of the UTAUT model. This study fills a gap in the application of the UTAUT model in the field of virtual reality for gastroscopy. Through this study, we hope to provide suggestions for the application of VR technology in the field of 3D and for educators to help medical students better accept VR gastroscopy technology.

## Materials and methods

### Sample

Using a stratified sampling method, we invited master’s and doctoral students majored in clinical medicine in 5 comprehensive universities and 5 medical universities in China to participate in our research project through an electronic questionnaire, during February and March of 2021. Considering the response rate, we set up an incentive mechanism. Subjects who complete the questionnaire can get a reward of a red envelope. This study was reviewed and approved of by the Ethics Committee of Health Culture Research Center, the key research base of Philosophy and Social Science in Shaanxi province because the survey was anonymous and we declare that all personal data collected will be treated strictly confidential in the Informed Consent Form (ICF) on the first page of the questionnaire. The signing of the ICF was voluntary, if the students refuse to sign the ICF, they were deemed to have withdrawn from the research. A total of 347 students were invited to complete the survey, with a response rate of 99.14% (344), of which 52 responses were excluded: 20 were excluded due to response time less than 5 min, 14 for duplicate IP addresses, and 18 for failing the logic check. A total of 292 responses were used for further analysis, so the effectivity rate is 84.15%.

### Survey questionnaire

The questionnaire contains 2 parts. The first part contains the basic information of the participants, including gender, university name, grade, clinical study time and hometown. The second part is based on the UTAUT model, using a 5-point Likert scale, including 5 dimensions and 19 questions(1 = strongly disagree, 2 = disagree, 3 = neutral, 4 = agree, and 5 = strongly agree). The composition of the questionnaire adapted from the literature is shown in Table 1.

**Table 1:** Measurement Constructs and Items

Construct	Items
Performance Expectancy	Using the 3D gastroscopic model to study can improve my manipulative ability [30].
	Using the 3D gastroscopic model to study can enable me to have a more thorough grasp of theoretical knowledge [31].
	Using the 3D gastroscopic model to study can improve my learning quality [32].
	Using the 3D gastroscopic model to study can provide more learning resources for me to complete important courses [30].
	Using the 3D gastroscopy model to study is conducive to the development and cultivation of my independent learning ability [33].
	Using the 3D gastroscopic model can improve my learning efficiency [32].
Effort Expectancy	I think I can easily learn how to operate the 3D gastroscopic model [34].
	I think it won’t take me long to be skilled in using the 3D gastroscopy model [30].
	I think learning how to use the 3D gastroscopic model is not difficult for me [35].
Social Influence	I think my teacher will recommend me to use the 3D gastroscopy model [31].
	I think my school/college will support the use of 3D gastroscopy model [36].
	I think my schoolmates will recommend me to use the 3D gastroscopy model [31].
	If most of my classmates use the 3D gastroscopic model for learning, I will use it too [37].
Facilitating Conditions	When I encounter difficulties in using the 3D gastroscopy model to learn, I have teachers or classmates who can help me solve relevant problems for help [30].
	I have the knowledge of how to use the 3D gastroscopic model [30].
	I have relevant resources to learn how to use 3D gastroscopic model [30].
Behavioural Intention	I am very interested in the 3D gastroscopic model and would like to know more [38].
	I have some understanding of similar VR models, and may try to use the 3D gastroscopic model to learn [38].
	I think I will try to use the 3D gastroscopic model to learn [36].

In order to improve the validity of the questionnaire, we chose a pilot study from the study population. The fundamental purpose of the pilot study is to empirically verify the reliability of the questionnaire by examining the accuracy and veracity of entire measurement constructs and items. For respective construct, reliability was examined through Cronbach’s alpha, the threshold of which was set to 0.7 [39]. In the pilot study, we collected 50 eligible questionnaires from respondents in two universities. The Cronbach’s alpha scores ranged from 0.829 for FC to 0.933 for PE. According to the results of the Cronbach’s alpha values, all variables were above 0.7. The content validity of the questionnaire was evaluated according to the viewpoints of six specialists, including clinical medicine, public health, medical pedagogy, psychology, behavioristics and statistics. The reliability and usability of the final questionnaire was proved.

### Data analyses

IBM SPSS Statistics 25.0 (Network Version from Peking University, Address: 162.135.134.153) software was used to establish a database for statistical description and to do analytical statistics (Pearson’s correlation coefficient and Rank-sum test) to analyze the results. Using Amos 21.0 software, the convergent validity and discriminant validity of the measurement model were tested by confirmatory factor analysis (CFA) and all the hypothetical paths of the SEM were analyzed. PLS-SEM was also used to test discriminant validity of the measurement model. The maximum likelihood robust method was used to estimate the parameters. Significance was set to P < 0.01. The commonly used evaluation standards of SEM are Chi-square/degrees of freedom(X2/df), goodness-of-fit index (GFI), comparative fit index (CFI), root mean square error of approximation (RMSEA), normal fit index (NFI), and Tucker–Lewis index (TLI), adjusted goodness of fit index (AGFI), incremental fit index (IFI) [40]. All methods were carried out in accordance with relevant guidelines.

## Result

### Respondents

The collected sample comprises of 170 (58.2%) female and 122 (41.8%) male, the distribution of which was close to the gender ratio of the whole medical student population at China Medical Universities of 60.1% females versus 39.9% male students [38]. Among them, 145 (49.7%) of the students were from comprehensive universities and 147(50.3%) were from medical universities, and the distribution was close to the ratio of the entire comprehensive universities with medical schools versus medical universities in China of about 1 to 1. Also in this sample, 225 (77.1%) were master students and the rest 67(22.9%) were doctoral students. Moreover, less than half of the students (45.9%) were from rural China, suggesting that their family economic situation is relatively low compared with students in urban areas [41]. The details of the respondents’ demographic information are shown in Table 2.

**Table 2:** Basic information of the respondents

Items	Description	Number	Percentage (%)
Gender	Man	122	41.8
	Woman	170	58.2
Universities	Comprehensive universities	145	49.7
	Medical universities	147	50.3
Educational degree	Master student	225	77.1
	Doctoral students	67	22.9
Year of clinical learning	Less than 1 year	132	45.2
	1–2 years	81	27.7
	More than 2 years	79	27.0
Home location	Urban areas	158	54.1
	Rural areas	134	45.9

### Measurement model evaluation

Firstly, the common method variance was tested using Harman’s one-factor test and the result of 36.827% was lower than the threshold 40%, indicating that there is no serious common method bias in the scale used in this study. Before reporting the structural model, the collinearity value should be noted by reporting the variance inflation factor (VIF) values. In Table 3, all VIFs are found to be lower than 3, indicating that there are no significant multicollinearity problems in the model.

**Table 3:** Assessment of the SEM model

Construct	No. of items	Item loading	Cronbach’s α	AVE	CR	VIF
Performance Expectancy (PE)	6	0.81–0.88	0.94	0.715	0.938	1.129
Effort Expectancy (EE)	3	0.81–0.88	0.87	0.695	0.872	1.689
Social Influence (SI)	4	0.67–0.82	0.85	0.589	0.851	1.791
Facilitating Conditions (FC)	3	0.67–0.86	0.82	0.628	0.833	1.249
Behavioural Intention (BI)	3	0.79–0.84	0.85	0.666	0.857	

AVE, Average Variance Extracted; CR, Composite Reliability

We assessed the measurement model by examining the internal reliability, convergent validity (CV), and discriminant validity (DV). The internal reliability was assessed by checking the Cronbach’s alpha and composite reliability (CR) values for every construct. CV was evaluated by measuring the average variance extracted (AVE). See Table 3 for the results of the item loading range, Cronbach’s alpha, AVE, and CR.

In Table 3, the calculated construct loadings range from 0.67 to 0.88, all exceeding the recommended levels [39]. Construct reliability, which indicates how well a construct is measured by its items, was assessed through Cronbach’s alpha and composite reliability (CR). The Cronbach’s alpha values ranged from 0.82 for FC to 0.94 for PE, and CR values ranged from 0.833 for FC to 0.938 for PE. For both measures, all constructs exceeded the recommended cutoff of 0.7 [42], suggesting high internal reliability. As is shown in Table 3, the estimated latent construct factor loadings ranged from 0.67 to 0.88 and were statistically significant (*p* < 0.05). The AVE ranged from 0.589 (SI) to 0.715 (PE) and exceeded the threshold of 0.5 for each construct [42], suggesting high convergent validity.

To assess the DV, the square root of the AVE of each latent construct was compared with its inter-construct correlation. The square root of the AVE of a construct should exceed its correlations with other constructs to achieve appropriate DV [42, 43]. Moreover, the diagonal values should exceed the off-diagonal values in the corresponding columns and rows [44]. Table 4 shows that for every construct, the square root of the AVE (bold values shown diagonally) was higher than the inter-construct correlations, thus indicating a satisfactory level of DV.

**Table 4:** Fornell-Larcker criterion (FLC).

Constructs	PE	EE	SI	FC	BI
PE	**0.846**				
EE	0.182*	**0.834**			
SI	0.348*	0.721*	**0.767**		
FC	0.215*	0.459*	0.464*	**0.792**	
BI	0.427*	0.311*	0.481*	0.491*	**0.816**

SD, Standard deviation; Bolded values on the diagonal are the square root of the AVE Values on the off-diagonal represent inter-construct correlations. SI, Social Influence; PE, Performance Expectancy; EE, Effort Expectancy; FC, Facilitating Conditions; BI, Behavioural Intention. *p < 0.05.

However, if used in combination with results of variance-based structural equation modelling such as traditional partial least squares path modeling and generalized structured component analysis, the Fornell-Larcker criterion lacks sensitivity, and if used in combination with consistent estimates, it lacks specificity [45]. Thus, besides using the Fornell-Larcker criterion, we also used the heterotrait-monotrait ratio of correlations (HTMT) which is a novel approach for assessing discriminant validity introduced by Henseler, Ringle and Sarstedt to make sure the test of discriminant validity is rigor [46]. If the HTMT is smaller than one, discriminant validity can be regarded as established. In many practical situations, a threshold of 0.85 reliably distinguishes between those pairs of latent variables that are discriminant valid and those that are not. The HTMT was calculated using the PLS-SEM software package and also the results were shown in Table 5.

**Table 5:** Heterotrait-Monotrait Ratio (HTMT).

Factors	PE	EE	SI	FC
EE	0.184			
SI	0.357	0.714		
FC	0.258	0.457	0.466	
BI	0.425	0.313	0.481	0.531

SI, Social Influence; PE, Performance Expectancy; EE, Effort Expectancy; FC, Facilitating Conditions; BI, Behavioural Intention.

### Relationship between the demographic characteristics moderators and latent variables

In most cases, the relationship between variables was not significant (p > 0.05). It was found that there was a significant correlation between gender and effort expectancy (p < 0.05). Moreover, facilitating conditions had significant correlation with universities (p < 0.01) or education degree (p < 0.01) of the respondents. The correlation between behavioural intention and hometown (p < 0.05) or duration of clinical learning (p < 0.01) of the respondents was also quite significant.

### Hypothesis testing

The quality of the SEM was assessed by examining fitness indexes of the model and variance-explained estimates. The fitness indexes of the model are shown as follows: X2/df was 2.385 < 3, GFI = 0.885>0.8, NFI = 0.908>0.9, TLI = 0.934>0.9,CFI = 0.944>0.9,IFI = 0.944 >0.9, AGFI = 0.849>0.8 and RMSEA = 0.069 < 0.08, which demonstrates that the hypothesis model in this research was supported (See Table 6 for details).

**Table 6:** Fitness indexes of the model

Index	Calculated result	Ideal result
RMSEA	0.069	< 0.08
Chi-square	2.385	1–3
CFI	0.944	> 0.9
NFI	0.908	> 0.9
TLI	0.934	> 0.9
IFI	0.944	> 0.9
AGFI	0.849	> 0.8
GFI	0.885	> 0.8

All hypotheses were supported in this study, except hypothesis H5 stating that effort expectancy positively affects behavioural intention (β=-0.112, p = 0.240).The standardized path coefficient of the structural model were presented in Table 7.The results show that the relationships between performance expectancy and behavioural intention (β = 0.275, p < 0.01), social influence and behavioural intention (β = 0.313, p < 0.01), facilitating condition and behavioural intention (β = 0.331,p < 0.01) were significant. Thus, H3, H4, and H6 were confirmed.

**Table 7:** Hypothesis testing

Hypothesis	Path	Estimate	*P*	Decision
H1	SI→PE	0.340*	< 0.001	Supported
H2	SI→EE	0.727*	0.000	Supported
H3	PE→BI	0.275*	0.000	Supported
H4	SI→BI	0.313*	0.004	Supported
H5	EE→BI	-0.112	0.240	Not supported
H6	FC→BI	0.331*	0.000	Supported

SI, Social Influence; PE, Performance Expectancy; EE, Effort Expectancy; FC, Facilitating Conditions; BI, Behavioural Intention. * Significant at P < 0.01

**Fig. 2:**
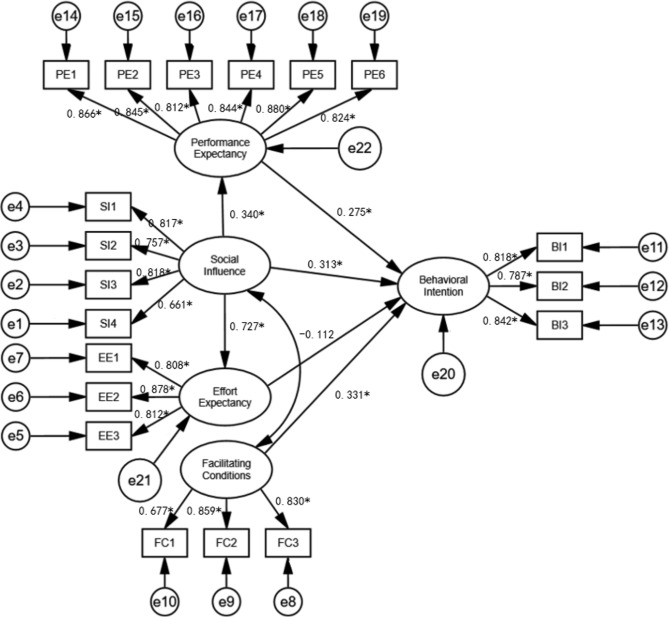
Path coefficients for the research mode. SI, Social Influence; PE, Performance Expectancy; EE, Effort Expectancy; FC, Facilitating Conditions; BI, Behavioural Intention. *P < 0.01

In addition, a significant relationship between social influence and performance expectancy (β = 0.340, p < 0.01), social influence and effort expectancy (β = 0.727, p < 0.01), were observed outside the standard UTAUT model. Therefore, H1 and H2 were confirmed. The overview of the standardized path coefficient of the structural model were shown in Fig. 2.

## Discussion

### The influence of respondents’ general characteristics on students’ behavioural intention to use 3D gastroscopic model constructed based on VR technology

In this study, we tried to explore how variables affect students’ behavioural intention to use 3D gastroscopic model constructed based on VR technology between the students studying in the university of clinical medical sciences. Results showed that there was a significant correlation between year of clinical learning and students’ behavioural intention of using the 3D gastroscopic model based on VR technology. Through postmortem analysis, we confirmed that students who had studied in the clinic for one year and one to two years showed a stronger willingness to use the technology than those who had studied in the clinic for more than two years. There was no significant difference in behavioural intention between students who had studied in the clinic for one year and one to two years. Perhaps because students who had studied in the clinic for more than two years are more experienced in the operation of gastroscopy as a result of longer years of study and practice. The same explanation applied to the significant effect of educational degree on facilitating conditions that master students perceive more technique or knowledge support from teachers or organizations like universities than doctoral students [47].Besides, on the contrary of the findings of Venkatesh et al. that the moderating effect of gender on effort expectancy to behavioural intention will be stronger for women, we found that the score of male students’ effort expectancy is higher than that of female students with a significance.

Different from the stratification of types of universities and colleges key universities and colleges (KUC) and non-key universities and colleges (NKUC) in the research of Hongbin Wu,et al. [42], we used comprehensive universities and medical universities as the basis for stratification because they have different educational environment and mode of teaching which may influence the behavioural intention of the students. For comprehensive universities, supported by humanities and social sciences and other natural sciences, the multi-level cultivation of accomplishment, ability and knowledge are emphasized, so that medical students can develop higher overall accomplishment and comprehensive strength on the basis of good professional skills. For medical universities, the central work of the school serves the cultivation of medical talents and the main activities of the school are carried out around the cultivation of medical students’ medical practice skills, which is conducive to the cultivation of medical professionals but relatively lack of humanistic environment [48]. The results of the rank-sum test showed that there was a significant difference in the scores of facilitating conditions between the students from different kinds of universities, with higher scores in students of medical universities, proving our stratification to be reasonable. Considering that few previous studies have taken the types of university or college into consideration, these results make a unique contribution to the literature [41].

In addition, home location was found as a key moderating variable in this research that affected the behavioural intention of using VR technology among students. In our research, 143 medical students from rural areas of China have weaker behavioural intention to use the 3D gastroscope model based on VR technology than those from urban areas. We speculate that the reason for this difference is that there is a big gap between urban and rural areas in China [49]. The family economic status of medical students in rural areas is relatively lower than that of students in urban areas [50], and they are slow to accept new things or technologies. This is consistent with a study on online patient services, which found that there were differences between rural and urban residents in using patient portals [51].Also, this is consistent with previous research findings and the study by Ma Q et al. [52] that economic status was strongly associated with the acceptance of a technology in developing countries.

### The influence of the dimensions of the structural equation model based on UTAUT on students’ behavioural intention to use 3D gastroscopic model constructed based on VR technology

Traditional gastroscopy teaching has many limitations, and it is difficult to take into account the risks of patients and the actual operation of students [53–55].VR technology provides a better teaching method for gastroscopy teaching, which can provide a very real environment for gastroscopy operation and diagnosis for students and improve the operation ability of gastroscopy [56–58]. The study adopted the theoretical framework of original and extended UTAUT model aimed to explore the acceptance of 3D gastroscopic model constructed based on VR technology by clinical medical students and the factors influencing their willingness to inoculate themselves against this new teaching modality.

Four determinants in the research model were theorized according to the acceptance model but the findings were not perfectly fitted as hypothesized. The results of the study confirmed that UTAUT could mostly predict students’ behavioural intention to use gastroscopic model constructed based on VR technology. Regarding UTAUT correlation variables, the result shows that three variables of performance expectancy, facilitating condition, and social influence had a significant influence on the behavioural intention of using the 3D gastroscopic model constructed based on VR technology. This is consistent with our assumptions for H3, H4, H6.

Firstly, performance expectations significantly influenced medical students’ behavioural intention to receive the VR gastroscopy system, which suggests that the higher the medical students’ expectations that the VR gastroscopy system can help improve their medical skills, the stronger the acceptance of the VR gastroscopy system. Ali garavand et al. used the UTAUT model to analyze the factors influencing the adoption of mHealth applications among medical students concluded that performance expectations have a significant positive effect on behavioural intentions [23]. Yousef et al. concluded in a study using the UTAUT model to predict patients’ intention to use their personal health records that performance expectations were significantly correlated with behavioural intentions, consistent with our findings [59]. Secondly, social influence had a significant effect on medical students’ behavioural intention to receive the VR gastroscopy system. This indicates that the environment around medical students affects their behavioural intention towards VR gastroscopy system, such as teachers and classmates are supportive and favorable to VR gastroscopy system, which will increase medical students’ acceptance of VR gastroscopy system. In a study of the acceptance and influencing factors of virtual reality socialization among urban elderly people, the researchers analyzed through the UTAUT model and found that social influence and performance expectations are important factors affecting the acceptance of virtual reality socialization among elderly people [60]. When assessing human acceptance of AR-assisted assembly scenarios, Schuster et al. verified that social influence has a positive effect on behavioural intention [61]. This was also confirmed by the meta-analysis performed by Dwivedi et al., in which the influence of social factors on behavioural intentions was significant in most studies [18]. Finally, facilitating condition has a positive and most significant effect on behavioural intention in this study. This indicates that if medical students find the VR gastroscopy system easy to learn and use, their acceptance of the VR gastroscopy system will increase significantly. In the study based on extended UTAUT model for understanding the impact of trust on user acceptance of cloud computing, Saad Alharbi et al. [62]found that performance expectancy and facilitation conditions were the important influencing factors. The study of BrizPonce and García-Peñalvo [63] and Schomakers et al. [64] found that the facilitating condition factor is a strong predictor that affects behavioural intention. These are all consistent with the results of this study.

In addition, we have verified the significance between social influence and performance expectation, social influence and effort expectation, thus the H1 and H2 hypotheses are verified. This is consistent with several studies done by scholars such as Paula Philippi and Mengting Chen [27, 28]. If students’ teachers or classmates recommend them to use the 3D gastroscopic model constructed based on VR technology, they will probably consider the technology easy to use, or the technology will help them attain gains in study performance, thus improve their acceptance of the technology. These two hypotheses are outside the UTAUT model, and few studies focus on the relationship between them. The positive results of our study implies that there is a need that future research should pay more attention to do more verification on the relationship between social influence and performance expectancy, as well as the relationship between social influence and effort expectancy.

According to the results of this study, effort expectancy plays insignificant effects on behavioural intention. However, previous studies have indicated that effort expectancy plays a significant effect on users’ behavioural intention to employ a fresh technology [65–67]. The startling inconsistency may be due to the situation that VR has yet to be widely adopted in medical education thus the students have poor familiarity with the 3D gastroscopic model constructed based on VR technology. If the students don’t have the idea of how to use or how to learn to use the 3D gastroscopic model constructed based on VR technology, they may be not sure whether they can work hard to get themselves skilled in this totally new thing. The assumption is relatively reasonable because previous studies have indicated that effort expectancy plays a significant effect on users’ behavioral intention focus on some Apps or techniques that have been widely used and respondents are familiar with them.

### Theoretical and practical contributions of this study

The theoretical contributions of this study are as follows: This study applies UTAUT model in the field of VR gastroscopy for the first time, and explores medical students’ behavioural intention to receive VR gastroscopy, filling the gaps in previous research fields such between UTAUT and virtual reality. In addition, this study adapted the UTAUT model framework to the original UTAUT model suggested by Venkatesh et al. in the context of this study. The four moderating variables of the original UTAUT model (gender, age, experience, and voluntariness of use) were adjusted to the five demographic factors of gender, type of college, grade level, year of clinical study, and home location. Also, the significance between social influence and performance expectancy, social influence and effort expectance which researchers didn’t pay much attention to were verified.

The practical contributions of this study are that its findings could help medical educators to better teach about the use of 3D gastroscopy models. This could help medical schools to make better use of the technology in teaching medical knowledge and improve students’ understanding and operational skills in relation to diseases, diagnosis and treatment, thereby reducing surgical risks and improving patient safety. In addition, this study could also provide a reference for similar improvements in teaching and the application of new technologies in other subject areas, as well as the development of teaching tools for related companies.

### Limitations

The limitations of the study are as follows. Firstly, as the research data is in the form of self-reporting, it is difficult to eliminate some recall bias. Secondly, this study is a cross-sectional design study, which does not capture causal inference. Therefore, it does not capture the factors that influence medical students’ behavioural intention to use VR Gastroscopic model, but rather factors related to phenomena. What’s more, the latent variable “use behaviour” were excluded from our model because of the fact that the traditional high simulation devices too expensive to be used widely in medical schools in China, so many of the respondents in our survey didn’t have the experience of using VR technology to learn. Future studies can recruit respondents from countries that have a common application of VR technology in gastroscopy teaching and explore the relationships between use behaviour and other variables.

## Conclusions

Compared with traditional gastroscopy teaching, applying VR technology in gastroscopy teaching has prominent advantages, for it can make up for the inherent defects of the traditional teaching mode. This study applied the UTAUT model to explain the determinants of clinical medical students’ behavioural intention toward using the 3D gastroscopic model constructed based on VR technology. Taking comprehensive universities and medical universities as the basis for stratification, data were collected from 292 respondents. We excluded the manipulated variable “experience” and “voluntariness”, and changed the “age” to “year of clinical learning”. At the same time, we brought in manipulated variables “home location” and “educational degree”, which offered fresh perspectives on the interaction between the four prior latent variables as they played effects on behavioural intention.

The most important conclusions are as follows: High internal consistency and reliability of the model were testified, hence manifesting sufficient explanatory power of the model based on UTAUT proposed in this study. Positive effects of performance expectancy, social influence and facilitating conditions on behaviour intention toward using the 3D gastroscopic model constructed based on VR technology were proved in this study. Furthermore, social influence is a key factor that plays prominent effects on effort expectancy of clinical medical students, and the results manifested positive effect of social influence on performance expectancy, which provided a direction for future studies.

An important variable of effort expectancy in the frame of the SEM were not certified, thereby indicating that particular attention should be paid to this variable by universities and teachers before applying 3D gastroscopic model constructed based on VR technology in teaching. Added preparatory work is required such as explaining the basic knowledge of the operating steps of VR model and make students adequately understand its accessibility, which can probably improve the intentions of them to use it. Additionally, we suggest that VR gastroscopy teaching should be integrated with the traditional teaching mode and the designed operating steps should be easy to learn and command. In particular, the instructors should be practiced in sorts of types of operations of VR gastroscopy teaching system.

## Data Availability

The authors declare that the supporting data can be obtained from the corresponding authors upon reasonable request.
